# Technical evaluation and optimization of a mobile septage treatment unit

**DOI:** 10.1016/j.jenvman.2020.111361

**Published:** 2021-01-01

**Authors:** Aaron A. Forbis-Stokes, Arumugam Kalimuthu, Janani Ravindran, Marc A. Deshusses

**Affiliations:** a Department of Civil & Environmental Engineering, Duke University, Durham, NC, USA; b Triangle Environmental Health Initiative, Durham, NC, USA; c Water, Sanitation and Hygiene Institute, Kodaikanal, India; d Duke Global Health Institute, Duke University, Durham, NC, USA

**Keywords:** Onsite wastewater treatment, Septage, Ultrafiltration, Microfiltration, Mobile, Full-scale

## Abstract

A mobile septage treatment unit was built in India using readily available filters and membranes (mesh fabric, sand, granular activated carbon (GAC), microfilter, ultrafilter) and installed on the bed of a small truck. The target application was emptying of septic or sewage holding tanks and concentration of suspended solids while generating a liquid that could be discharged. The system was evaluated for operational and treatment performance while processing septage in the field at 108 sites in Tamil Nadu, India. After one phase of evaluation (Phase I), the system was improved and three replicate systems with slight modifications were fabricated for a second round of evaluation (Phase II) alongside the original, but modified unit. In Phase I, 105 m^3^ of septage was processed at an average flow of 623 L h^−1^ and with high removal efficiencies: 83% chemical oxygen demand (COD), 75% total suspended solids (TSS), and 98.4% total coliform (TC). In Phase II, the original and three new systems combined treated 168 m^3^ of septage. One of the new systems doubled in capacity and processed septage at an average flow of 2700 L h^−1^ while the other three averaged 1290 L h^−1^. The removal efficiencies in Phase II were 80% COD, 81% TSS, and 99% TC averaged between the four systems. Pass through of soluble contaminants (e.g. soluble COD, NH_3_–N) remain the primary challenge for treatment performance. Success may be limited with some septage due to seasonality, location, or septage age, and further validation and optimization may be necessary. However, the septage in this study was treated to local standards, and the system offers a method of onsite treatment while reducing the need of costly and often inefficient septage emptying services. Further, the system can be produced at a cost competitive to traditional septage hauling trucks.

## Highlights

• A membrane treatment system was installed in a truck for mobile septage treatment.
• The system provided high level of treatment at a high flow meeting local standards.
• Capital cost of truck and system was comparable to trucks which only accept septage.
• The possibility to treat septage at its source decreases burden of transport.

## Graphical abstract

**Figure:**
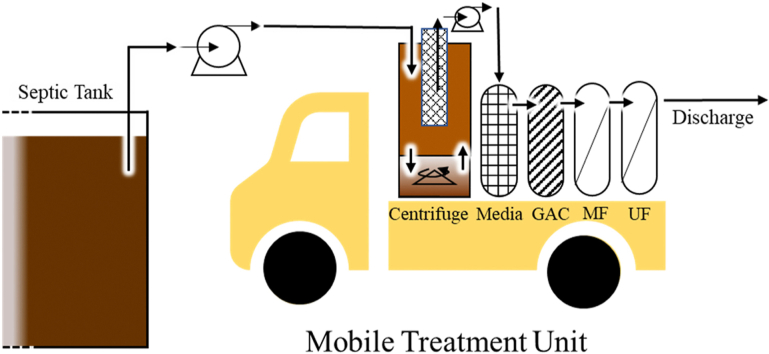

## 1 Introduction

The 2011 India census found that 38.2% of urban households with toilets are connected to septic tanks (Government of India, 2011). This percentage increases to greater than 62% for cities with less than one million residents. Under the Swachh Bharat Mission, 21% of already constructed toilets in rural households are connected to septic tanks, and 24% of those under construction will be connected to septic tanks (Water Aid, 2017). Based upon these findings, India's wastewater framework is heavily reliant on septic tank services. Septic tanks in India's urban and peri-urban regions typically do not have liquid overflows to soak pits or soil absorption fields, as seen in many other locations, but are large vaults that are emptied when full. Septic truck operators are hired to empty these tanks once full, with the purpose of transporting contents to tipping stations or sewage treatment plants. However, it is often found that septic contents do not arrive at these treatment stations. The distance to travel, fuel cost, time demand and expenses all motivate emptiers to dump their contents in alternative, nearby locations without providing sanitary treatment of the septic truck contents. Evidence of this indiscriminate dumping is seen in the global city-wide excreta flow diagrams that show as much as, or more than 50% of collected septage is not treated in cities of India or many other countries (SuSanA, 2020). Two excreta flow diagram examples in India include the major city of Chennai (population 7.1 million) and smaller city of Kochi (population 2.1 million). Chennai's excreta flow diagram shows 58% of population using onsite sanitation of which 66% requires emptying, and only 61% of that is treated (Narayan and Ramachandran, 2019). Meanwhile, 78% of Kochi's population uses onsite sanitation, of which only 14% is treated (Roeder, 2016).

Due to these issues, decentralized wastewater/sewage treatment has garnered greater attention lately (Capodaglio et al., 2017; Chirisa et al., 2017; Singh et al., 2015; UN-Habitat and Asian Institute of Technology (AIT), 2015). Typical approaches to decentralized treatment include low-tech approaches such as wetlands, lagoons, drying beds, scaled down version of wastewater treatment plants, etc. (Forbis-Stokes et al., 2020, 2016; Rogers et al., 2018). Membrane processes are increasingly used for wastewater treatment at centralized and decentralized facilities due to their ability to achieve high levels of treatment while utilizing a small footprint (Landsman et al., 2020). The technology developed and evaluated in this study, a mobile septage treatment unit (MTU), was designed to address the financial and logistical barriers to transport, so that septic tanks are emptied when needed and contents are properly treated before being released into the environment. The mobile treatment unit that was developed utilizes affordable filtration installed on the bed of a small truck to create a mobile approach to septage treatment.

A study of 240 septic tanks in Chennai, India, located in the same state where this technology was developed, found that local septic waste had the following characteristics reported as averages with standard deviation in parenthesis: 2870 (2320) mg TS L^−1^, 907 (1430) mg TSS L^−1^, 1182 (1050) mg COD L^−1^, 382 (400) mg soluble COD (sCOD) L^−1^, 76 (65) mg L^−1^ total nitrogen (TN), and 66 (41) mg PO_4_^−^ L^−1^ (Krithika et al., 2017). The study also found that suspended solid and organic concentrations were 1.6 times higher in winter months (November–February) compared to summer months (March–May). Prior particle size distribution research of septic tank effluent found a range of 0.1–1000 μm with the majority falling between 1 and 100 μm (Troesch et al., 2009). In light of these properties, the multistage process of the MTU was designed to treat septage with a series of filters decreasing in filtration pore sizes to incrementally remove contaminants along the particle size distribution, down to less than 0.02 μm. This design was expected to remove all suspended material and some soluble material. Granular activated carbon (GAC) was also used to help with removal of soluble material including organics and nutrients such as ammonia. In terms of pathogens, bacteria should be removed at the microfiltration level based on pore size, and ultrafiltration provides additional security. As an indicator of virus removal, previous work with MS2 coliphage found microfiltration to have an average log reduction value (LRV) of 1.38 (95% confidence interval (CI) was 0.70–2.07) (Amarasiri et al., 2017; Iranpour, 1998; Jolis et al., 1999) while ultrafiltration membranes were found to have an average LRV of 3.69 (95% CI 2.87–4.52) for MS2 coliphage (Amarasiri et al., 2017; Frohnert et al., 2015; Lu et al., 2013). Combining these elements was expected to produce an effluent without suspended contaminants and greatly reduced in soluble and pathogenic contaminants, possibly meeting local discharge standards.

The primary goals for the MTU were to (1) empty septic waste and treat its contents onsite to meet India's treatment standards for discharge, (2) treat the waste at a high rate (between 2000 and 3000 L h^−1^) so that multiple tanks could be treated per system each day, and (3) meet the first two goals using materials and methods that require low capital and operational expenditures.

## 2 Materials & methods

The MTU was evaluated in two study periods hereon named Phase I and Phase II. Phase I evaluation provided baseline knowledge of MTU capabilities and lasted from October 2017 to February 2018. Findings from Phase I provided considerations for improved MTU design. During Phase II, four MTUs (the original one modified, and three newly constructed and improved ones) were evaluated in different locations from March to May 2018.

### 2.1 System materials

**Fig. 1:**
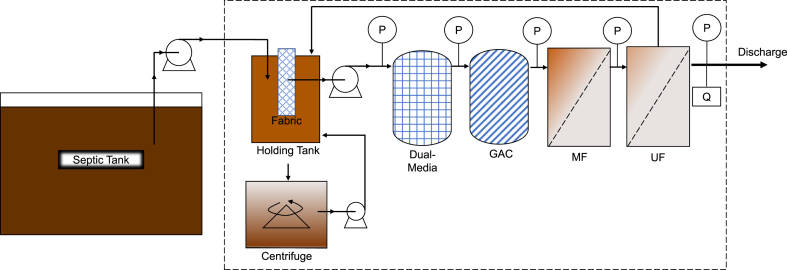
Mobile treatment unit (MTU) process flow diagram with a dashed line designating which materials are located on the MTU truck. Symbols “P” and “Q” designate locations of pressure and flow gauges, respectively. Septage is pumped from a household septic tank into a holding tank through a fabric-covered feed tube. Liquid is pumped from the holding tank through the series of filters: Dual-Media (D–M), GAC, MF, and UF. UF effluent is discharged into environment, while only the concentrated solids are transported to a centralized treatment system. The UF reject value is partially opened to allow some reject to return to the holding tank, shown by arrow returning from UF to holding tank. Solids settled in the holding tank flow to centrifuge while centrate is returned to the settling tank. Figure is not to scale.

The MTU systems were built on the bed of 2-ton trucks (Mahindra “Bolero Maxi-Truck,” Mumbai, India). Total weight of each truck after additional equipment was 3.1 tons. Fig. 1 displays a process flow diagram consistent for all MTUs. The following details describe the initial MTU (thereafter MTU-1) and are valid for each new MTU except for where specified. A 0.5 HP mono-block centrifugal pump (Texon Engineering, Coimbatore, India) drew waste from the septic tank using a 5 cm internal diameter (ID) hose pipe inserted through one of the septic tank's risers. This liquid was sent into a 500 L holding tank located on the truck. The middle of the holding tank contained a 25 cm ID PVC pipe, 90 cm in height, with 10 mm diameter (dia.) holes drilled into the top 75 cm of the pipe to allow septic tank supernatant to fill the pipe. The holes covered this upper section with 10 mm spacing between each. The outside wall of that pipe was wrapped with a #250 mesh (58 μm) fabric to pre-filter the septic waste before pumping. The purpose of this fabric was to remove the larger particles and thereby extend the life of the succeeding filters by using a low-cost and easy to clean material. From the center of this pipe, liquid was pumped using a 1 HP mono-block, double capacitor centrifugal pump (CRI Pumps, Coimbatore, India), inducing an outside-in filtration of septage from holding tank into the fabric-covered pipe. The next filter was a dual-media (D-M) filter housed in a 190 L (166 cm height, 41.2 cm dia.) fiber-reinforced plastic (FRP) container. Flow entered the FRP container at the bottom and exited from the top. The bottom 60 cm was filled with large pebbles (30–60 mm dia.), the next 45 cm was filled with small pebbles (4–30 mm dia.), and the top 15 cm was filled with coarse sand (0.5–1.0 mm dia.). The remaining volume was left empty. The D-M filtrate then entered a granular activated carbon (GAC) (Krishna Industrial, Chennai, India) filter housed in a FRP container with the same dimensions as the D-M filter, containing approximately 85 kg of GAC of #4 × 8 mesh size (2.4–4.8 mm) with 1200–1800 m^2^ g^−1^ surface area. GAC filtrate entered two microfilter (MF) polypropylene-wound filter cartridges (Filtcare Technology, Ahmedabad, India) in series, both of 50 cm length and 11.4 cm dia. The membranes had a nominal pore size of 10 and 5 μm, respectively. MF effluent was treated with an ultrafiltration (UF) membrane (Vens Marketing, VM-200/1650 “The Way”, Chennai, India). The hollow fiber membranes had an outer dia. of 0.1 mm and nominal pore size of 0.02 μm with an outside-in flow direction. All filtrations except the UF were conducted as dead-end filtration. The bottom of the holding tank was connected to a centrifuge (1 HP, 2800 RPM, 30 L volume). The centrifuge concentrated solids that settled in the holding tank. Centrate (i.e., clarified liquid leaving the centrifuge) was returned to holding tank using a submersible pump.

For Phase II of the study, the process flow remained the same, but some designs were changed as follows. The holding tank was modified to have a conical-bottom to improve solids settling, and a baffle was installed around the feed pipe to better restrict the flow of solid particles towards the feed pipe. The D-M filter media was changed to the following media distribution (bottom to top): 30 cm large pebbles (30–60 mm dia.), 30 cm small pebbles (4–30 mm dia.), 30 cm coarse sand (0.5–1.0 mm dia.), 15 cm fine sand (0.125–0.250 mm dia), and 15 cm anthracite (1–2 mm dia.). The same type and quantity of GAC as in Phase I was used. The same FRP containers were used for the D-M and GAC filters as in Phase I. Larger MFs were used and the number of units was increased to increase total surface area of the filters. The first MF was 5 wound cartridges operating in parallel with nominal pore size of 10 μm, 76 cm length and 6.4 cm dia. The second MF was also 5 cartridges operating in parallel, 76 cm length and 6.4 cm dia. but with nominal pore size of 1 μm (Placon Filters, Chennai, India). The UF was replaced with a ZeeWeed 1500 UF with 55.7 m^2^ surface area, 192 cm length, 18 cm dia., and 0.02 μm nominal pore size (Suez Water Technologies, Trevose, PA, USA).

The modifications described above were made to the initial unit (MTU-1), and three additional units were fabricated: MTU-2a, MTU-2b, and MTU-2c. Each of these had slight modifications from the changes previously described. MTU-2a used a pre-screening filter placed between the septage pump and the holding tank to remove larger suspended particles >100 μm. MTU-2b had the septage pumped into the bottom of the conical tank and a 100 μm screen was placed above the influent to pre-screen and trap larger particles in the bottom of the tank. MTU-2c used two UFs in parallel and a centrifuge with twice the capacity to see if total flow and solids handling could be improved enough to justify greater capital expense.

### 2.2 Study location

In Phase I, the MTU was operated in the Dindigul district, located in the state of Tamil Nadu. The district covers 6000 km^2^ and has a population of 2.15 million according to the 2011 census. Of this population, 20.8% of the households are connected to septic systems. For Phase II, the study location included the Dindigul district and it was expanded into Madurai and Trichy districts. Madurai covers 3700 km^2^ with a population of 3.04 million. Trichy covers 4500 km^2^ with 2.72 million people. The additional locations were chosen to broaden the testing conditions while maintaining a reasonable proximity to Dindigul for research purposes.

### 2.3 Methods

Phase I analysis took place from October 2017 to February 2018, and Phase II took place from March to May 2018. Operational performance was analyzed by taking pressure, flow, and power readings every 15 min for the first hour and every 30 min for the remaining time of operation. Pressure readings were taken using 0–4 bar (±0.05 bar, Micro Process Controls EN 837-1, Gujarat, India) analog gauges which were located before and after each filter. The two MFs in series were considered as one MF unit for analysis. The transmembrane pressure was calculated as the difference in pressure between the two gauges and was used as an indicator of filter fouling. Discrete flow was measured using a 500-5000 L h^−1^ rotameter (ASTER Technologies F-5,000L*,* New Delhi, India) while total volume treated was measured using a water totalizer, 0–1.0 × 10^7^ L (±0.1 L, TKT Water Meters B1214436, Coimbatore, India). Power consumption was determined using a current meter. Power was supplied by the house being serviced.

Treatment performance analysis was completed by taking samples at each step in the process near the end of operation and analyzed in the Water, Sanitation, and Hygiene Institute (WASHi) laboratory. All sampling points were analyzed for COD (IS 3025-58) and turbidity (IS 3025-10). Raw septage and final effluent were additionally tested for pH (portable meter and probe), Biological Oxygen Demand (BOD_5_) (Trivedy and Goel, 1986), TSS (IS 3025-17), ammonia (NH_3_) (Trivedy and Goel, 1986), phosphate (PO_4_^3−^) (Trivedy and Goel, 1986), and total coliform (TC) (Standard Methods 9222B).

During Phase I, MTU-1 would typically treat one septic tank of 1000–3000 L per day. The system was operated manually, turning the septic and filtration pumps on and off as needed. The centrifuge was only used at the end of the treatment period for each septic tank because most homes could not provide sufficient power to run the septage supply and filtration pumps and the centrifuge at the same time. Therefore, the centrifuge was only used at the end when the septic pump was turned off. The UF was installed with a manually controlled reject line which returned retentate to the holding tank. The system was not backwashed while in operation but was backwashed with water at the end of the operation after treating each septic tank. The system was chemically washed with NaOCl (200 ppm) and NaOH (500 ppm), and then HCl (500 ppm) once a month.

During Phase II, treating two septic tanks per day was targeted. Operation was manual (as during Phase I). The UF partial reject flow was decreased, and a reverse flow backwashing protocol and schedule were initiated. After every 30 min of operation, forward pumping was stopped, and collected UF effluent was pumped in the reverse direction for 30 s. Backwash liquid returned to the holding tank. The centrifuge was also used during this period in an attempt to achieve more solids removal and allow for higher flow rates during normal operation. Backwashing filters with water and rejecting the return continued to be done at the end of each day, and chemical washing was conducted as before (monthly with 200 ppm NaOCl, 500 ppm NaOH, and 500 ppm HCl).

## 3 Results

### 3.1 Wastewater characteristics

#### 3.1.1 Phase I

**Fig. 2:**
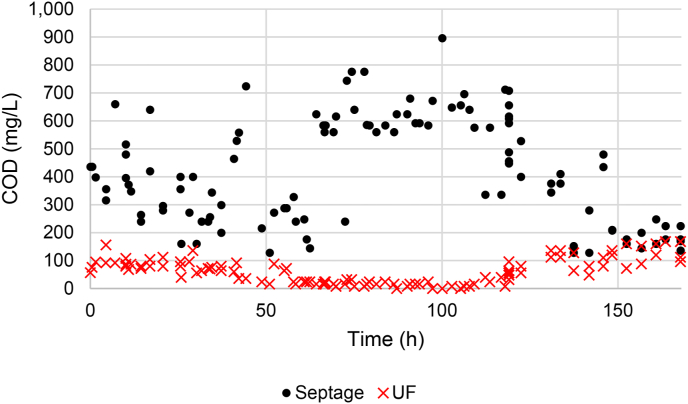
Measured COD of the raw septage and UF (final) effluent measured according to total run time of septage treatment operation during Phase I. Typically each run lasted 2–4 h.

Table 1 displays results for wastewater characteristics from each step in the MTU. The right side of the table references the latest effluent discharge standards for India's sewage treatment plants (Ministry of Environment Forest and Climate Change, 2017). The most recent regulations include a different set of standards for metro (Mumbai, Delhi, Kolkata, Chennai, Bengaluru, Hyderabad, Ahmedabad and Pune) and non-metro areas, the latter of which is shown in the table, as that one better describes the target locations for the MTU. Table 2 displays the percentage removal performance of each filter stage in the MTU. The left side of the table shows the percent removal of each parameter for that filter's effluent with respect to the raw septic waste, while the right side displays the percent removal for that filter with respect to that filter's influent. As can be expected, the pH had negligible change through the process. The BOD (90% removal), COD (90%), TSS (76%), and TC (98.8%) all had high removal percentages from the influent to the final effluent. Though final effluent parameters had high standard deviation values relative to their averages (Table 1), final effluent variation did not correlate with septage concentrations (Fig. 2). For example, UF permeate concentrations were consistently low while starting septage concentration had high variations. Nutrients had the least amount of reduction through the system, 15% removal of TN and 47% removal of TP. The average values of BOD, COD, and TSS in the UF effluent began to increase after January 10, 2018 (i.e., after 102 h operation, see Fig. 2). At this point in the study, the UF had treated 63,500 L of waste in Phase I and 80,000 L of waste previously and may have surpassed its capacity. The average effluent BOD, COD, and TSS concentrations from October 2017 to January 10, 2018, were 19 mg_BOD_ L^−1^, 48 mg_COD_ L^−1^, and 37 mg_TSS_ L^−1^, which results in 92%, 89%, and 86% removal from the influent, respectively.

**Table 1:** Summary of average effluent concentrations from each step of the MTU-1 process during Phase I (N = 110). Unlike other parameters, TN and TC were not measured at every step (NA = not analyzed).

	Septage		Fabric filter		Dual-Media		GAC		MF		UF		Discharge
**Parameter**	**Avg.**	**St.Dev.**	**Avg.**	**St.Dev.**	**Avg.**	**St.Dev.**	**Avg.**	**St.Dev.**	**Avg.**	**St.Dev.**	**Avg.**	**St.Dev.**	**India Std.**
pH	7.7	0.6	7.9	0.6	7.9	0.5	7.8	0.5	7.8	0.5	7.7	0.5	**6.5–9**
BOD (mg/L)	217	94	82	51	58	30	42	25	35	22	24	19	**30**
COD (mg/L)	429	189	129	108	104	81	94	72	82	62	61	44	**N/A**
TSS (mg/L)	296	201	205	148	195	145	184	146	169	139	79	95	**100**
Turbidity (NTU)	75.7	40.1	52.9	28.7	50.2	27.2	46.4	27.0	42.1	26.0	18.3	20.1	**N/A**
TN (mg/L)	87.3	113.4	NA	NA	NA	NA	NA	NA	NA	NA	74.0	102.8	**N/A**
PO_4_ (mg/L)	4.9	2.2	3.9	1.9	3.4	1.7	3.1	1.6	2.9	1.6	2.6	2.0	**N/A**
TC (CFU/100 mL)	5300	5628	NA	NA	NA	NA	NA	NA	NA	NA	75	33	**1000**

**Table 2:** Average percentage removal, or percent change for pH, for each filter in MTU-1 based on the raw septage (left) or the inlet to that filter (right) for Phase I (N = 110).

		Total removal from septage					Removal based on each filter influent				
Parameter	Septage	Fabric	D-M	GAC	MF	UF	Fabric	D-M	GAC	MF	UF
pH	7.7	−2%	−2%	−1%	−1%	0%	−2%	0%	0%	0%	1%
BOD (mg/L)	217	62%	73%	80%	84%	89%	62%	29%	27%	17%	31%
COD (mg/L)	429	70%	76%	78%	81%	86%	70%	20%	9%	13%	25%
TSS (mg/L)	296	31%	34%	38%	43%	73%	31%	5%	5%	8%	53%
Turbidity (NTU)	75.7	30%	34%	39%	44%	76%	30%	5%	8%	9%	56%
TN (mg/L)	87.3	NA	NA	NA	NA	15%	NA	NA	NA	NA	NA
PO_4_ (mg/L)	4.9	21%	30%	37%	41%	47%	21%	11%	9%	7%	11%
TC (CFU/100 mL)	5300	NA	NA	NA	NA	99%	NA	NA	NA	NA	NA

The step percentage removal in Table 2 shows that the fabric filter was one of the most effective filters. It had the highest step removal of COD, BOD, and TP, and it was second only to the UF in TSS removal. The GAC was the least effective filter in the majority of parameters, but it did have a more significant removal of BOD.

Nitrogen speciation analysis during Phase I encountered some problems, as is often the case with heterogeneous samples with a high organic and high solids contents (Forbis-Stokes et al., 2020; Hunter and Deshusses, 2019). Though results were fairly consistent, the concentrations determined in-house did not match measurements from an outside laboratory (T.Stanes & Company, Coimbatore, India). For example, for one sample, the outside lab found a Total Kjeldahl Nitrogen (TKN) and NH_4_^+^ raw septic waste concentrations of 234 mg TKN L^−1^ and 191 mg NH_4_^+^-N L^−1^ compared to 6.2 mg TKN L^−1^ and 5.3 mg NH_4_^+^-N L^−1^ in the WASHi lab. The external lab's values were more in line with expected septage concentrations and were therefore used for performance analysis. Both labs reported negligible concentrations of NO_3_–N and NO_2_–N, meaning that TKN is well representative of TN. The outside lab reported final effluent concentrations of 145 mg TKN L^−1^ and 118 mg NH_4_^+^-N L^−1^, 38% removal for each TKN and ammonium.

#### 3.1.2 Phase II

Results from Phase II of this study are shown in Table 3 and Table 4. Table 3 shows raw septage and final effluent values from each MTU-2 while Table 4 shows the percent removal from septage to final effluent for the parameters analyzed. Only Phase I lab results are shown for MTU-1 as the Phase I sample size (N = 110) was much larger than in Phase II (N = 13), and Phase II results were not statistically different.

**Table 3:** Summary of average concentrations and standard of deviation from inlet (“Septage”) and outlet for each MTU in Phase II (Number of observations: MTU-2a = 24, MTU-2b = 18, and MTU-2c = 11). See Table 1 for threshold levels according to Indian discharge standards.

	Septage		MTU-2a		Septage		MTU-2b		Septage		MTU-2c	
Parameter	Avg.	St.Dev.	Avg.	St.Dev.	Avg.	St.Dev.	Avg.	St.Dev.	Avg.	St.Dev.	Avg.	St.Dev.
pH	7.7	0.5	7.8	0.5	7.8	0.4	7.9	0.5	7.8	0.3	7.8	0.3
BOD (mg/L)	193	178	23	8	148	85	35	27	711	773	27	11
COD (mg/L)	1000	1530	157	98	253	176	100	23	2510	3330	177	97
TSS (mg/L)	2010	2029	381	450	809	529	264	194	4400	4760	308	177
Turbidity (NTU)	604	2070	27	43	173	211	21	27	744	940	20	42
NH_3_ (mg/L)	152	99	141	80	256	185	173	181	142	95	104	57
PO_4_ (mg/L)	14	13	14	18	8	7	5	1	35	38	15	13
TC (CFU/100 mL)	59,800	95,500	437	778	8300	8900	127	45	456,000	598,000	1450	1870

**Table 4:** Percent removal, (or percent change for pH), for each MTU for its effluent in reference to the raw septage for that MTU during Phase I for MTU-1 and Phase II for all MTU-2.

	Total removal			
**Parameter**	**MTU-1**	**MTU-2a**	**MTU-2b**	**MTU-2c**
pH	0%	−1%	−2%	0%
BOD (mg/L)	89%	94%	77%	96%
COD (mg/L)	83%	84%	60%	93%
TSS (mg/L)	75%	90%	67%	93%
Turbidity (NTU)	72%	96%	88%	97%
NH_3_ (mg/L)	15%	7%	37%	58%
PO_4_ (mg/L)	45%	0%	37%	58%
TC (CFU/100 mL)	98.4%	99.3%	98.5%	99.7%

The percent removal performance of MTUs in Phase II was similar to Phase I with a few exceptions. MTU-2c (two UFs in parallel) had significantly higher removal of organics and solids, the highest of all units. MTU-2a & MTU-2b had worse COD removal (Table 2 vs. Table 4). However, the inlet COD concentrations for MTU-2a and MTU-2c in Phase II were higher than in Phase I (429, 1,000, 253, and 2510 mg COD L^−1^ for MTU-1, MTU-2a, MTU-2b, and MTU-2c, respectively) while inlet TSS concentrations where higher for all MTUs in Phase II (296, 2,010, 809, and 4400 mg TSS L^−1^ for MTU-1, MTU-2a, MTU-2b, and MTU-2c, respectively).

### 3.2 Operational characteristics

#### 3.2.1 Phase I

**Fig. 3:**
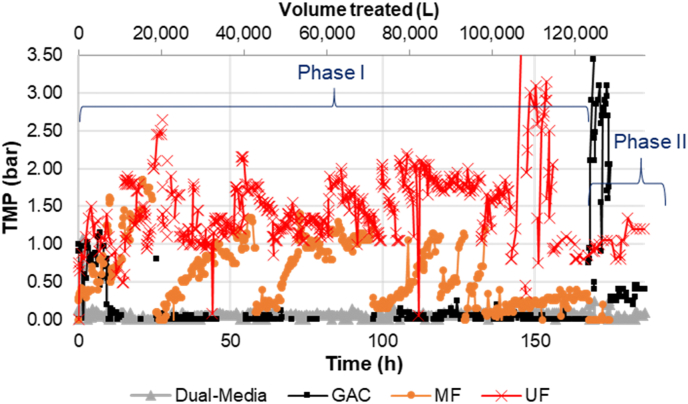
Transmembrane pressure (TMP) of each filter as a function of MTU-1 operational time during both Phases I and II.

The MTU in Phase I was operated for a total cumulative time of 170 h split between about 57 emptying operations lasting 2–4 h each. Transmembrane pressure (TMP) values for each filter are shown in Fig. 3. The D-M and GAC TMP values remained low for the majority of the study. The rise of TMP for the MFs was quite rapid, as seen from 0 to 26 h, 26–58 h, 58–79 h, and 97–125 h. During some of these increased pressure periods for the MF, the TMP for the MF was greater than the TMP for the UF. The TMP of a MF membrane was expected to be about one tenth of a UF membrane, thus our values indicate that the MF had severe fouling issues during these periods. The MFs were replaced after 25.6 h operation (18,000 L treated), 58.3 h (38,300 L), 96.9 h (60,300 L), 118.0 h (73,300 L), 127.0 h (77,600 L), and 131.0 h (79,600 L). The MFs treated approximately 18,000; 20,300; 22,000; 13,000; 4300; and 2000 L of waste, respectively. The last three replacements were not due to necessity but for experimentation. The MF septage treatment volume capacity based on this study was on average 20,000 L with 258 mg TSS L^−1^, or a total of 5.16 kg TSS.

A bag filter was added between the GAC and MF on December 27, 2017, (79.4 h; 50,700 L) to help extend the life of the MFs. Starting from February 5 (122.4 h) the MFs were chemically washed with NaOCl and NaOH for 20 min and then HCl for 20 min after each day of use. The filter bag did not appear to greatly improve performance as the TMP did not decrease from 79.4 to 96.9 h, and the rate of TMP increase with the new MF after 96.9 h did not decrease. The daily chemical washing that began after 122.4 h, however, did significantly impact MF performance. The TMP rate of increase was only 0.4 bar over 40 h, compared to 1.0 bar within 20 h without the washing.

**Fig. 4:**
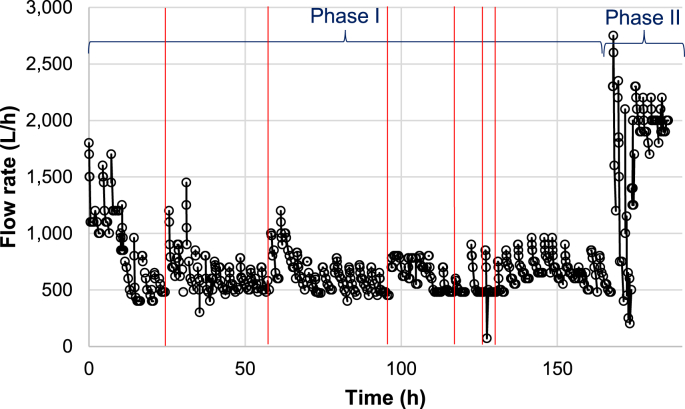
MTU flow rate over total operational time. Red vertical lines denote MF replacement events. (For interpretation of the references to colour in this figure legend, the reader is referred to the Web version of this article.)

Flow measured at the outlet during the operation time is shown in Fig. 4. The average flow during Phase I was 716 L h^−1^ (with a standard deviation of 234 L h^−1^). Because the filters were backwashed at the end of the day after each day of operation, the flow was generally high at the start of the day and decreased quickly over the next few hours while operating. There was also a general trend for the maximum flow which decreased from approximately 1700 to 600 L h^−1^ after 20 h of operation. The MF TMP increased rapidly over this same time period (Fig. 3), and the flow increased back up to 1500 L h^−1^ when the MF was replaced. Again, the flow quickly decreased until the MF was replaced after 58.3 h of operation. The combined results from TMP and flow suggest that the MF was the primary factor restricting the operational flow in the MTU system for the first 60 h of operation. The MF was replaced three more times after the 60 h mark, but the flow did not increase a significant amount after these replacements. Simultaneously, the general trend of the UF TMP was increasing. It may be interpreted that the UF membrane was the primary component restricting flow after 60 h of operation.

Operational experience showed that the flow of incoming septage was often higher than the filtration capacity due to fouling of membrane surfaces. Fouling of the fabric filter around the filtration feed tube would cause the inner portion of the tube to empty more quickly than the incoming septage could fill it, thus risking to run the feed pump dry and the 500 L tank to overflow. Therefore, the septic pump was manually turned off and on frequently during the treatment period to equalize flows. The fouling rate of the fabric filter was magnified when treating septic tanks with higher solids content which led to the feed tube lifting out of holding tank. Under this condition, the filter pump was turned off temporarily in order for the septic waste to reenter the feed tube. Based upon this experience, the holding tank was redesigned for Phase II to improve solids separation in the septic waste tank. The standard water tank was replaced with a conical-shaped tank to improve solids settling, and a baffle wall was wrapped around the feed pipe to increase the flow path of incoming waste to the fabric filter. Each of these modifications appeared to have mitigated these issues; however, no controlled tests were performed to determine which provided the greatest benefit.

The average current draw for MTU operation was 9.0 A at 220 VAC (single-phase). Assuming power factor of 1, the operating power was 1.98 kW. The average operation time was 3.49 h (with a standard deviation of 0.93), resulting in 6.9 kWh energy demand per septic tank treated. Grid power outages were frequent in the test region. The MTU operated on power supplied by the home being serviced, so these power outages stopped MTU operation, causing delays of up to 30 min per outage. Additionally, periodic power shut-downs scheduled by local governments for daytime hours prevented the MTU from operating during those days. A portable generator may be desirable for future MTUs so that these external issues do not interfere with proper operation.

#### 3.2.2 Phase II

**Fig. 5:**
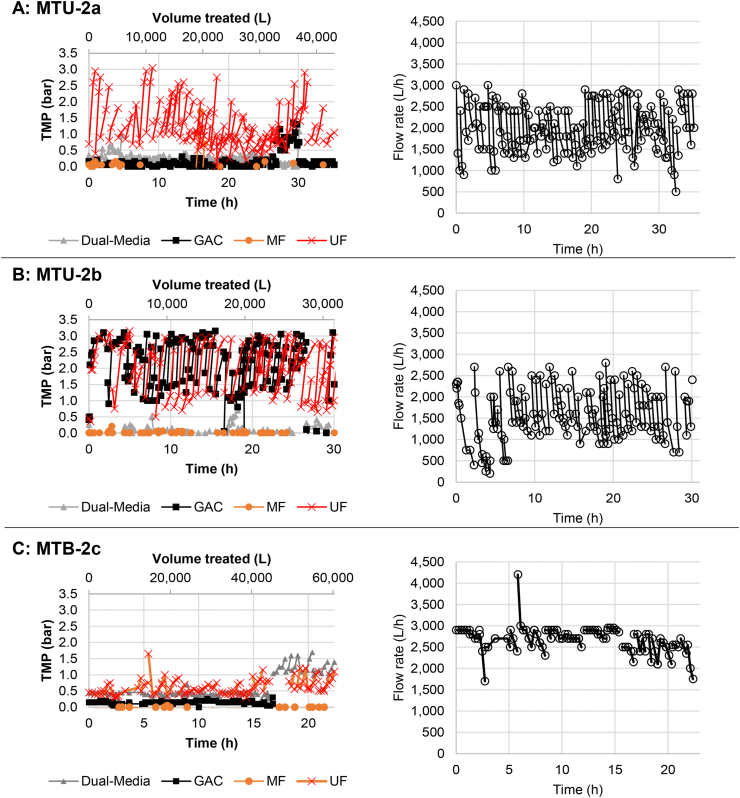
Transmembrane pressure (TMP) and flow rate for each filter vs. the total MTU operating time (lower x-axis) and total volume treated (upper x-axis). Note that x-axes are all on different scales due to different operating times.

Fig. 5 displays the TMP and the flow rate over time for all MTU-2 (see Fig. 2, Fig. 4 for Phase II results for the MTU-1). The results showed improved reduction in UF TMP between cycles. Whereas TMP for the UF quickly increased and remained at a high level during Phase I (Fig. 3), the UF TMP in Phase II increased but fell back to a stable level with regular flushing (Fig. 5). Additionally, the MF TMP did not increase over time as had been observed with MTU-1 during Phase I. These two findings indicate the improved MF materials prevented them from fouling as quickly as during Phase I, and the backwashing protocol prevented, or at least significantly delayed, irreversible fouling of the UF.

Flow rate values from each MTU in Phase II were all improved. During Phase I, after only 10 h of operation, peak flow rates never exceeded 1500 L h^−1^, and average flow rates were less than 1000 L h^−1^. During Phase II, all MTUs maintained an average flow greater than 1500 L h^−1^, with more than 20 h of operation in each system. Further, no system displayed a downward trend in peak or average flow rate while MTU-1 in Phase I showed a downward trend almost immediately.

Though the dual-UF system in MTU-2c provided higher flow rates, they are not double the flow rates of other systems. Approximately 4000 L h^−1^ was expected if there was a linear relationship between UF size and flow rate. Pump capacity may have been the limiting factor for flow rate in MTU-2c instead of UF capacity as the TMP of the double UF system rarely exceeded 1 bar.

## 4 Discussion

### 4.1 Wastewater treatment effectiveness

Based on these results, the MTU in Phase I (MTU-1) met India Discharge standards for all parameters which called for effluent having less than 30 mg_BOD_ L^−1^ and 100 mg_TSS_ L^−1^ (Ministry of Environment Forest and Climate Change, 2017). Under the newest regulations for discharge standards, Metro areas have more strict standards for BOD and TSS (20 vs. 30 mg_BOD_ L^−1^ and 50 vs. 100 mg_TSS_ L^−1^). As discussed in Section 3.1, if analysis was stopped at January 10, 2018, the average concentrations of BOD and TSS in the effluent would have been 19 mg_BOD_ L^−1^ and 37 mg_TSS_ L^−1^. The MTU effluent would then have met effluent standards even under the strictest conditions of the current standards. The current Indian discharge standards do not consider COD, turbidity, TN, or TP. However, past standards included limits of 100 mg_COD_ L^−1^ and 10 mg_TN_ L^−1^. These targets were removed due to the difficulty for wastewater treatment plants to achieve them. Even so, maintaining a high level of treatment is desired in the event that any future regulations include more strict limits. The MTU effluent was well below 100 mg_COD_ L^−1^. The total nitrogen concentration did decrease from inlet to outlet (typically 87 down to 74 mg_TN_ L^−1^), but not sufficiently to meet the older strict standards. The TN removal observed was assumed to be mostly the organic fraction of N removed by filtration of organic particles. Though consistent and reliable ammonia analysis was a challenge, the results indicated that the majority of total nitrogen was in the form of NH_4_^+^, which is expected for aged septic waste. NH_4_^+^ removal through adsorption onto GAC is a potential method of TN reduction in the existing MTU, but there is lacking evidence that adsorption occurred (no significant difference of TN concentration between inlet and outlet of GAC filter). GAC has a potential ammonia adsorption capacity of about 17 g kg^−1^ (Long et al., 2008). Based on this capacity, the MTU with 85 kg of GAC could have removed 1.46 kg of NH_3_. If 90% of the average concentration of 32.3 mg TN L^−1^ (the concentration measured by WASHi's lab) in the raw septic was total ammoniacal nitrogen (TAN) (29 mg TAN L^−1^), the MTU could have removed TAN from 50,000 L. However, if the outside laboratory's results for NH_4_^+^-N were used for the inlet TAN (191 mg NH_4_^+^-N L^−1^), the MTU could have removed TAN from only 7600 L of septic waste. This volume could result in a requirement of replacing the GAC every two days if two tanks are treated per day, which is not a realistic option for MTU operation. Depending on the true amount of NH_4_^+^ in the septic waste, and what, if any, nutrient limit is imposed upon the MTU effluent, additional nitrogen management solutions may be necessary.

The COD adsorption capacity of the GAC was also be considered. The fabric filter and dual-media filter provided the bulk of COD removal (82% of total influent COD removed at this point) because most COD was suspended matter. The majority of COD remaining could be considered mostly soluble with some fine suspended COD. Previous adsorption studies using GAC to remove COD from blackwater (urine, feces, and flush water), found adsorption capacities of 34 and 48 g_COD_ kg_GAC_^−1^ treating inlet concentrations of 1100 and 1200 mg_COD_ L^−1^, respectively (Huggins et al., 2016; Rogers et al., 2018). If the GAC in our study had a similar COD adsorption capacity of 40 g_COD_ kg_GAC_^−1^ and the D-M effluent averaged 83 mg_COD_ L^−1^, the 85 kg of GAC could then remove COD from 40,900 L of waste. However, based upon results from this study, an average of only 7.9 mg_COD_ L^−1^ was removed from the total flow of 104,500 L. That removal resulted in a removal capacity of only 9.7 mg_COD_ g_GAC_^−1^. The BOD removal, though, was higher. An average of 16.1 mg_BOD_ L^−1^ was removed in the GAC filter, a removal capacity equivalent to 19.8 mg_BOD_ g_GAC_^−1^. Both of these removal capacities are much lower than expected. One reason for low adsorption in the GAC filter could potentially be related to GAC quality. The GAC was purchased from a local supplier who may not have had sufficient quality control. Another possibility is fouled external surfaces may have prevented availability to the internal porous structure. Placing the GAC filter after the MF or UF should be considered in the future, as it would limit direct deposition of solids onto the GAC.

Both the GAC and MF did not exhibit a high removal rate for most parameters. At this point in the treatment process, the filtrate has passed through a #250 mesh fabric (58 μm) and a dual-media filter so that what remains is either fine particles and/or dissolved contaminants. The GAC did little to remove fine particles, and as discussed, the GAC provided minimal removal of soluble contaminants via adsorption, making the GAC filter one of the least effective. Though the MF did not display much removal, the MF fouled quickly and caused the most operational and maintenance burden. Based on rapid MF fouling, increasing the membrane surface area of the MF was done for Phase II of the study. Another approach that could be considered is switching the MF and GAC in the treatment process. Because the GAC provided little pre-treatment for the MF, then moving the MF upstream of the GAC should have minimal detrimental impact on MF performance. In return, the GAC would be assured to only receive dissolved materials which may improve its treatment capacity. Still, even if their efficacies were less than expected, the GAC and MF likely expanded the lifetime of the UF (treating 143,500 L before exceeding discharge standards).

The centrifuge did not provide a noticeable difference between the septage and centrate in terms of wastewater characteristics. However, the centrifuge removed suspended material and decreased the fouling rate of the fabric filter. Operational experience based on system response to high solids suggested that the settled sludge section of the septic tank was typically less than 5% of the total septic tank volume. The solids retained in the centrifuge were removed at the end of each day. Typically, less than 1 kg wet solids were removed per site from the average emptying volume of 1800 L. WASHi used the solids at their own facilities to make fuel briquettes. The solids can also be used for composting. Fabric centrifuge bags were used in this study, but biodegradable centrifuge bags (if available) could eliminate the need for any handling hazardous material by composting the entire bag. Other centrifuge models may not require a fabric or textile bag.

Results from Phase II indicated that the modified MTUs maintained similar percent removal of contaminants while treating waste at higher flow rates. However, some final effluent parameters were above Indian discharge limits (see Table 3). Average contaminants concentrations in septage increased from Phase I (winter) to Phase II (summer) for most parameters (see Table 3 vs. Table 2), which contradicts the experience from Krithika et al. (2017) who found that winter concentrations were higher in Chennai. Their finding may only be specific to Chennai. While the MTUs in Phase II had similar removal percentages as MTU-1 in Phase I while treating septage with higher concentrations, improvements are needed to ensure the final effluent is always meeting discharge limits. Further, the decrease in COD removal in MTU-2b was troubling. The changes made to the systems should have only improved performance, so there is no clear reason for the reduced COD removal. Soluble COD (sCOD) was not measured in this study due to lab constraints but would have been a valuable parameter. One possible reason for the decreased COD removal in Phase II is that the makeup of the COD in septic tanks had proportionally more sCOD and was not removed in final step of UF. The Chennai study by Krithika et al. (2017) also showed seasonality effects on the proportion of sCOD/COD and that the ratio in summer months (0.41) was higher than winter months (0.33). If that were true of the region at large, the change in seasons may have increased the soluble fraction. Another possibility is that sCOD/COD could change related to septage age, and Phase II MTUs were used to treat tanks of different ages than Phase I. This area should be investigated in future studies.

In terms of flow rates, the backwashing protocol implemented in Phase II was likely the main contributor to maintaining high flow rates. The goal of this protocol was to flush particles off the UF membrane while in operation to make operation more efficient during uptime and prevent or delay irreversible fouling. Though treatment was interrupted by this protocol, total treatment time was reduced due to the increased flow rates. For example, each new MTU treated more than twice the quantity of the initial MTU in the same amount of time. In addition to backwashing, the improved MFs and UFs had also increased treatment capacity and were less subject to fouling over time.

### 4.2 Lifespan, cost, and acceptability

The lifespan of most filters was beyond the duration of the study. Only the fabric filter and MF membranes were replaced during Phase I. The fabric filter was replaced monthly as it was found that it began to tear from abrasion, but the very low cost of the fabric made this replacement a non-issue. The MF membranes were replaced approximately every 20,000 L. As the MTU is intended to treat high volumes of wastewater per day (possibly up to 4000–6000 L d^−1^), the lifespan of the MF was much shorter than desired. Based upon this finding, it was determined that MF surface area needed to be increased for Phase II. MF cartridges were both of 50 cm length and 11.4 cm dia. The manufacturer was not able to provide the total membrane surface area for filters used during both Phase I and II, but the outer surface area for each MF apparatus in Phase I was 1790 cm^2^. For Phase II, each MF apparatus was replaced with 5 wound cartridges operating in parallel of 76 cm length and 6.4 cm dia. Each cartridge had an outer surface area of 1530 cm^2^, thus the total surface area of the 5 cartridges in parallel was 7640 cm^2^. The second MF was also 5 cartridges of 76 cm length and 6.4 cm dia. operating in parallel. Because the MF required the greatest amount of maintenance and appeared to cause the greatest restriction of flow, the expanded MF surface area was expected to reduce maintenance requirements and allow for a greater flow rate. This hypothesis was supported as the MF did not cause any operational challenges during Phase II, as previously discussed.

In Phase I of the study, the (2017) costs of the components were as follows: ₹600 ($9 US) for fabric filter mesh, replaced monthly over 4 months at ₹150 each; ₹3500 ($54 US) for D-M filter (media) and ₹9400 ($145 US) for the FRP container; ₹16,500 ($254 US) for GAC and ₹9400 ($145 US) for the FRP container, ₹650 ($10 US) for MF filter cartridges and housing, and ₹97,350 ($1500 US) for UF filters and housing for a total of 134,900 or about $1800 US. In Phase II of the study, the costs of the components were as follows: ₹150 ($2 US) for fabric filter mesh; ₹15,800 ($243 US) for D-M filter and container; ₹29,400 ($453 US) for GAC filter and container; ₹45,900 ($706 US) for MF filters and housing with each replacement cartridge at ₹190 ($3 US); and ₹141,600 ($2180 US) for UF filters and housing. The final cost of filtration-related components was ₹235,000 or $3600 US. The truck used and structural modifications to hold treatment components was ₹606,000 ($9317 US), pump hardware was ₹170,000 ($2600 US), and monitoring devices were ₹8600 ($100 US). The total cost of the MTU then was ₹1,020,000 or about $15,700 US. A typical septage emptying truck with vacuum pump in India costs $20,000–30,000 US. The MTU approach could, thus, empty and treat septage at a cost less than the standard emptying vehicle.

Working with thick septic sludge was rare during the five months of Phase I operation. The low solids and organic concentrations found are typical for Indian septic tanks due to high water usage. However, very thick sludge was encountered in two tanks while piloting the Phase II systems, which could not be successfully handled because the septic feed pump could not provide sufficient flow. One was a well-drained leach pit, and the other was a septic tank that allowed liquid to overflow and had not been emptied in possibly 15 years. One tank had a TSS concentration of 26,200 mg_TSS_ L^−1^. These characteristics were atypical and outside of the scope for which the MTUs were designed (lined septic tanks without effluent overflows), and the homeowners were not aware of their tank characteristics prior to the system's attempted treatment. Millions of low-solids septic tanks need emptying and treatment in India; however, future MTU versions or other systems should take these higher solids tanks into account.

No formal social acceptability studies of the MTU have been performed at this time. The primary concerns for social acceptance are odors associated with the treatment process and the aesthetics of the discharge liquid. Based on observation, odors were only noticeable within 1–3 m downwind of the truck. This odor can be classified as a noticeable fecal odor that is bearable or unpleasant, at worst, and rarely intense enough to encourage a bystander to move away. Electronic odor alert systems could be installed if deemed necessary (Kawadiya et al., 2020). The main odor source was the holding tank for the septage which had air gaps allowing odors to escape. A preliminary test in which the air openings were covered with a compost-biochar mixture removed all noticeable odors from the system. Regarding effluent aesthetics, the flowing discharge liquid appeared clear with a slight yellow tint and no detectable odor. This odor was only noticeable within 10–20 cm of collected effluent and was an earthy, musty scent that was not unpleasant.

## 5 Conclusion

Based upon the findings from Phase I, MTU effluents met the goal for India sewage treatment plant discharge standards. If more strict standards were applied, the greatest barrier would be nutrient removal, particularly nitrogen. During Phase II, the focus was placed on increasing throughput so that multiple tanks could be treated each day. The primary barriers to maintaining a high flow rate were fouling of the fabric filter and of the MF. For Phase II, better solids separation was implemented into the septage holding tank which reduced the fabric filter fouling rate. Secondly, the MF was replaced by multiple MFs of much greater surface area, which decreased the fouling frequency. Finally, a new backwashing protocol was developed and implemented. The downtime required for this backwashing was minimal in reference to normal operation time. The procedure was simple and allowed the MTU to maintain higher flow rates and for a longer period of time. The costs of these changes were low compared to the total MTU cost. However, these changes allowed for greater throughput so that more tanks could be treated in a shorter period of time, increasing potential income while not significantly increasing costs. Overall, the MTU has achieved or is close to achieving all of its goals. Subsequent to this study, the MTU was already put into valuable use. After severe flooding in the neighboring state of Kerala in August 2018, four MTU units were sent to provide disaster relief for flood victims. The MTUs treated 330,000 L of septage in a 10 day period. This use case is evidence of the benefit of this mobile and high-rate septage treatment system.

## Credit author statement

**Aaron A. Forbis-Stokes**: Conceptualization, Methodology, Formal analysis, Investigation, Data Curation, Writing - Original Draft, Writing - Review & Editing, Visualization. **Arumugam Kalimuthu**: Conceptualization, Methodology, Resources, Investigation, Writing - Review & Editing, Supervision, Project administration, Funding acquisition. **Janani Ravindran**: Investigation, Data Curation. **Marc A. Deshusses**: Conceptualization, Methodology, Resources, Writing - Original Draft, Writing - Review & Editing, Supervision, Project administration, Funding acquisition.

## Declaration of competing interest

The authors declare the following financial interests/personal relationships which may be considered as potential competing interests: Aaron Forbis-Stokes and Marc Deshusses declare that they have no known competing financial interests or personal relationships that could have appeared to influence the work reported in this paper. Arumugam Kalimuthu and Janani Ravindran are with the Water, Sanitation and Hygiene Institute, of Kodaikanal, India which is national level non-governmental organization. The WASH Institute is interested in the commercialization of the mobile treatment units presented and discussed in the paper.

## References

[bib1] Water Aid (2017). Quality and Sustainability of Toilets: A Rapid Assessment of Technologies under Swachh Bharat Mission - Gramin 24.

[bib2] Amarasiri M., Kitajima M., Nguyen T.H., Okabe S., Sano D. (2017). Bacteriophage removal efficiency as a validation and operational monitoring tool for virus reduction in wastewater reclamation: Review.

[bib3] Capodaglio A.G., Callegari A., Cecconet D., Molognoni D. (2017). Sustainability of decentralized wastewater treatment technologies. Water Pract. Technol..

[bib4] Chirisa I., Bandauko E., Matamanda A., Mandisvika G. (2017). Decentralized domestic wastewater systems in developing countries: the case study of Harare (Zimbabwe). Appl. Water Sci..

[bib5] Forbis-Stokes A., O'Meara P., Mugo W., Simiyu G., Deshusses M. (2016). Onsite fecal sludge treatment with the anaerobic digestion pasteurization latrine. Environ. Eng. Sci..

[bib6] Forbis-Stokes A., Miller G.H., Segretain A., Rabarison F., Andriambololona T., Deshusses M.A. (2020). Nutrient removal from human fecal sludge digestate in full-scale biological filters. Chemosphere.

[bib7] Frohnert A., Kreißel K., Lipp P., Dizer H., Hambsch B., Szewzyk R., Selinka H.-C. (2015). Removal of surrogate bacteriophages and enteric viruses from seeded environmental waters using a semi-technical ultrafiltration unit. Food Environ. Virol..

[bib8] Government of India (2011). Census of India.

[bib9] Huggins T.M., Haeger A., Biffinger J.C., Ren Z.J. (2016). Granular biochar compared with activated carbon for wastewater treatment and resource recovery. Water Res..

[bib10] Hunter B., Deshusses M.A. (2019). Resources recovery from high-strength human waste anaerobic digestate using simple nitrification and denitrification filters. Sci. Total Environ..

[bib11] Iranpour R. (1998). Virus removal by advanced membrane filtration for wastewater reclamation. Water Environ. Res..

[bib12] Jolis D., Hirano R., Pitt P. (1999). Tertiary treatment using microfiltration and UV disinfection for water reclamation. Water Environ. Res..

[bib13] Kawadiya S., Welling C., Grego S., Deshusses M.A. (2020). Fecal malodor detection using low-cost electrochemical sensors. Sensors.

[bib14] Krithika D., Thomas A.R., Iyer G.R., Kranert M., Philip L. (2017). Spatio-temporal variation of septage characteristics of a semi-arid metropolitan city in a developing country. Environ. Sci. Pollut. Res..

[bib15] Landsman M.R., Sujanani R., Brodfuehrer S.H., Cooper C.M., Darr A.G., Justin Davis R., Kim K., Kum S., Nalley L.K., Nomaan S.M., Oden C.P., Paspureddi A., Reimund K.K., Rowles L.S., Yeo S., Lawler D.F., Freeman B.D., Katz L.E. (2020). Water treatment: are membranes the panacea?. Annu. Rev. Chem. Biomol. Eng..

[bib16] Long X., Cheng H., Xin Z., Xiao W., Li W., Yuan W. (2008). Adsorption of ammonia on activated carbon from aqueous solutions. Environ. Prog..

[bib17] Lu R., Mosiman D., Nguyen T.H. (2013). Mechanisms of MS2 bacteriophage removal by fouled ultrafiltration membrane subjected to different cleaning methods. Environ. Sci. Technol..

[bib18] Ministry of Environment Forest and Climate Change (2017). Sewage Treatment Plants Effluent Discharge Standards.

[bib19] Narayan A., Ramachandran K. (2019). SFD Lite Report.

[bib20] Roeder L. (2016). SFD Promotion Initiative: Kochi, India, Final Report.

[bib21] Rogers T.W., Rogers T.S., Stoner M.H., Sellgren K.L., Lynch B.J., Forbis-Stokes A.A., Stoner B.R., Hawkins B.T. (2018). A granular activated carbon/electrochemical hybrid system for onsite treatment and reuse of blackwater. Water Res..

[bib22] Singh N.K., Kazmi A.A., Starkl M. (2015). A review on full-scale decentralized wastewater treatment systems: techno-economical approach. Water Sci. Technol..

[bib23] SuSanA (2020). SFDs Worldwide [WWW Document]. SFD Promot. Initiat. https://sfd.susana.org/about/worldwide-projects.

[bib24] Trivedy R.K., Goel P.K. (1986). Chemical and Biological Methods for Water Pollution Studies.

[bib25] Troesch S., Liénard A., Molle P., Merlin G., Esser D. (2009). Treatment of septage in sludge drying reed beds: a case study on pilot-scale beds. Water Sci. Technol..

[bib26] UN-Habitat, Asian Institute of Technology (AIT) (2015). Policy Guidance Manual on Wastewater Management with a Special Emphasis on Decentralized Wastewater Treatment Systems.

