# Supplementary material for: Technical evaluation and optimization of a mobile septage treatment unit
Source: J Environ Manage. 2021 Jan 1;277:111361. doi: 10.1016/j.jenvman.2020.111361 (PMC7695683; doi:10.1016/j.jenvman.2020.111361)
Supplement: Multimedia component 1 [file mmc1.docx]

# Supplementary Information for on-line publishing

**Technical Evaluation and Optimization of a Mobile Septage Treatment Unit**

Aaron A. Forbis-Stokes^a,b^, Arumugam Kalimuthu^c^, Janani Ravindran^c^,
Marc A. Deshusses^a,d*^

^a^ Department of Civil & Environmental Engineering, Duke University, Durham, NC

^b^ Triangle Environmental Health Initiative, Durham, NC, USA

^c^ Water, Sanitation and Hygiene Institute, Kodaikanal, India

^d^ Duke Global Health Institute, Duke University, Durham, NC, USA

**Corresponding author*: Department of Civil & Environmental Engineering, Duke University, 127C Hudson Hall, Box 90287, Durham, NC 27708, USA.

*Phone:* (919) 660-5480; *Fax*: (919) 660-5219.

*Emails:* [marc.deshusses@duke.edu](mailto:marc.deshusses@duke.edu) , [aaron@triangle-environmental.com](mailto:aaron@triangle-environmental.com)


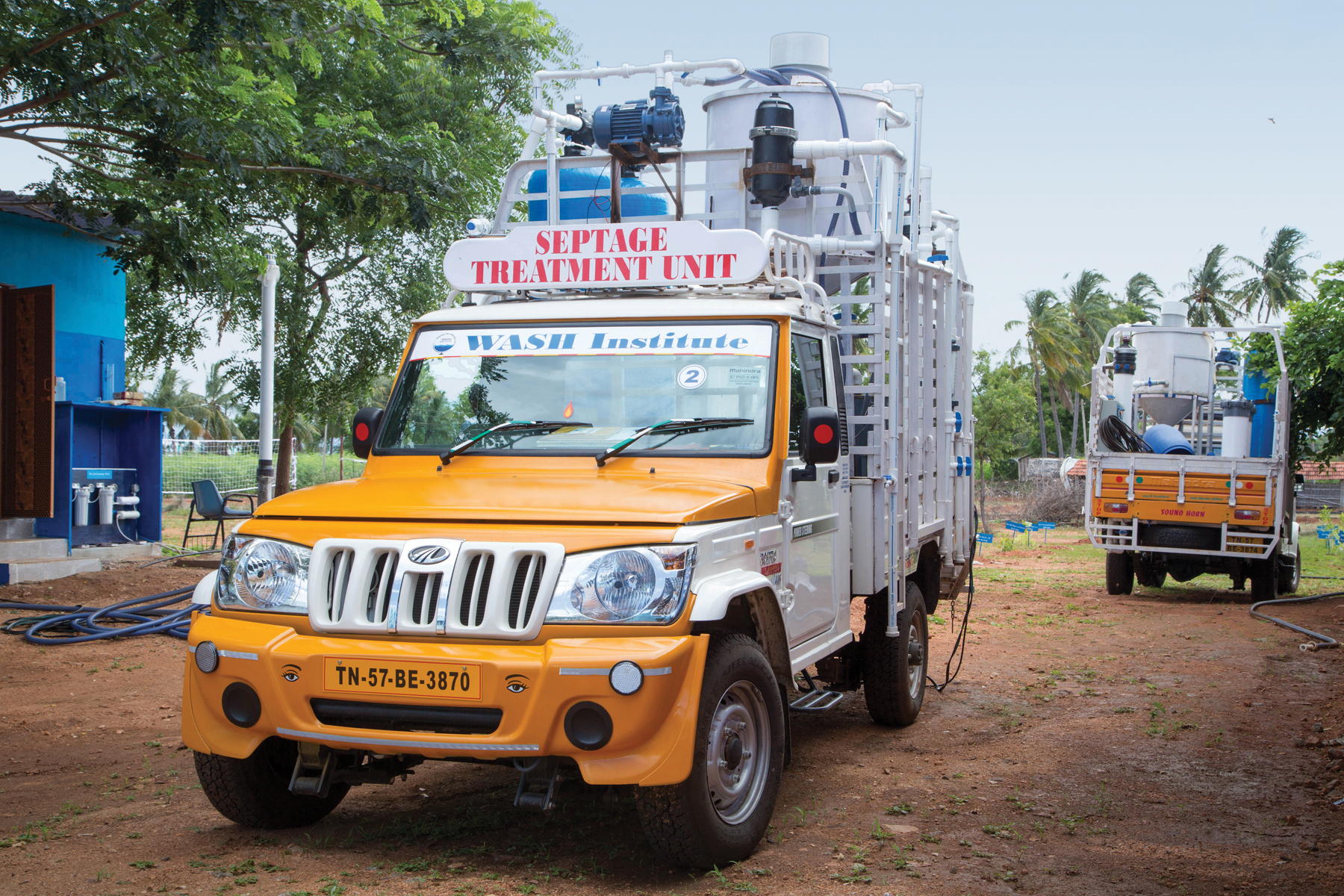


**Figure S1.** View of Mobile Treatment Units MTU-2b in front and MTU-2a in the background.


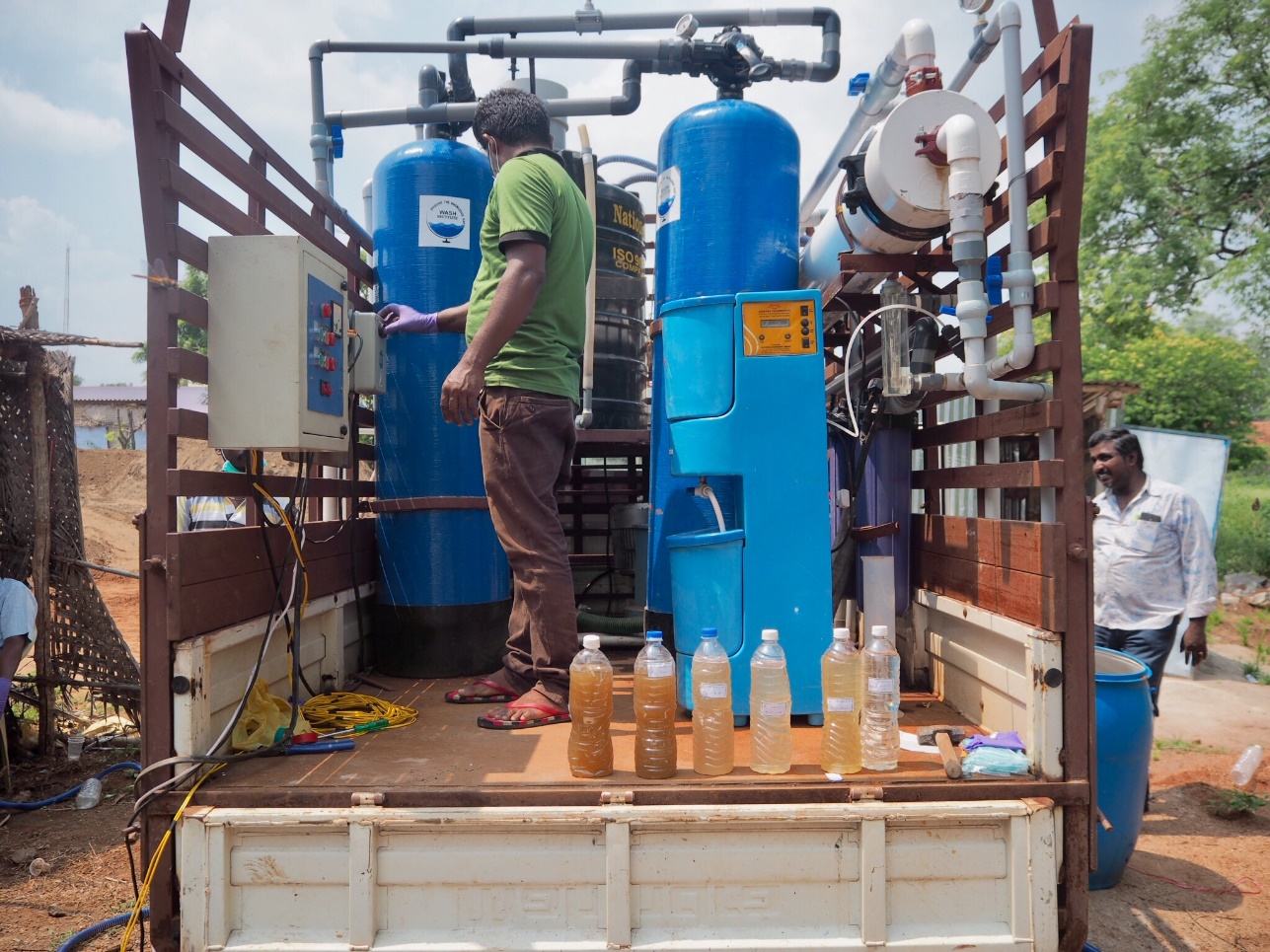


**Figure S2.** View of MTU-1 from rear with operator. Plastic bottles are samples taken along the treatment train: (from left to right) Septage, Fabric filter effluent, Dual-Media effluent, GAC effluent, MF effluent,
and UF effluent
